# Does a restricted energy low glycemic index diet have a different effect on overweight women with or without polycystic ovary syndrome?

**DOI:** 10.1186/s12902-019-0420-1

**Published:** 2019-09-02

**Authors:** Farnaz Shishehgar, Parvin Mirmiran, Maryam Rahmati, Maryam Tohidi, Fahimeh Ramezani Tehrani

**Affiliations:** 1 Department of Midwifery, Faculty of Nursing and Midwifery, Tehran Medical Sciences, Islamic Azad University, Tehran, Iran; 2 Nutrition and Endocrine Research Center, Research Institute for Endocrine Sciences, Shahid Beheshti University of Medical Sciences, Tehran, Iran; 3 School of Public Health, Department of Epidemiology and Biostatistics, Tehran University of Medical Sciences, Tehran, Iran; 4 Prevention of Metabolic Disorders Research Center, Research Institute for Endocrine Sciences, Shahid Beheshti University of Medical Sciences, Tehran, Iran; 5 Reproductive Endocrinology Research Center, Research Institute for Endocrine Sciences, Shahid Beheshti University of Medical Sciences, No 24, Parvane Street, Yaman Street, Velenjak, Tehran, Iran

**Keywords:** Polycystic ovary syndrome (PCOS), Diet, Obesity

## Abstract

**Background:**

Obese women with polycystic ovary syndrome (PCOS) may face additional barriers in achieving weight loss. We aimed to compare the effects of the hypocaloric low glycemic index (LGI) diet on anthropometric variables and insulin resistance in women with and without PCOS and investigate the effect of this diet on the clinical and hormonal features of PCOS women.

**Methods:**

This interventional study was carried out at the Reproductive Endocrinology Research Center, Research Institute for Endocrine Sciences, Shahid Beheshti University of Medical Sciences, Tehran, Iran. Of 108 women invited for the purpose of the present study, 62 participants (PCOS = 28, non-PCOS = 34) followed a 24-week energy restricted LGI diet. Anthropometric, biochemical, hormonal and clinical measurements were documented at baseline, 12 weeks and 24 weeks with intervention.

**Results:**

The percentages of weight loss achieved by both the PCOS and non-PCOS groups did not differ significantly (PCOS: -8.04% vs. non-PCOS: -8.09%). No significant difference in decrease of homeostatic model assessment of insulin resistance (HOMA-IR) was observed between the two groups (PCOS = − 0.83 ± 0.33, non PCOS = − 0.79 ± 0.28, *P* = 0.83). In PCOS women, significant reduction in total testosterone (− 0.91 ± 0.33 nmol/L, *P* = 0.006), FAI (− 4.47 ± 1.1, *P* < 0.001) and increase in SHBG (38.98 ± 11.02 nmol/L, *P* < 0.001) were observed. Menstrual irregularity was improved in 80% of women with PCOS and a significant decrease (32.1%) in occurrence of acne was reported.

**Conclusions:**

This diet has equally beneficial effects on anthropometric and metabolic characteristics of overweight women with and without PCOS.

**Trial registration:**

This study is registered in the Iranian Randomized Clinical Trials Registry (IRCT, code: IRCT2016092129909N1).

## Background

Polycystic ovary syndrome (PCOS) is the most common endocrinopathy in women of reproductive age; its pooled prevalence varies from to 7 to 12% based on the various criteria used for its definition [1]. The pathogenesis of PCOS is complicated and has not been clearly elucidated. Although obesity is not the leading cause of PCOS, it aggravates insulin resistance and endocrine and reproductive abnormalities particularly menstrual irregularities and hyperandrogenism [2]. As a result, decreasing insulin resistance has been targeted for PCOS management [3].

In a general population, the energy restricted low glycemic index (LGI) diet has led to greater improvement in insulin resistance and glucose metabolism [4]. Evidence show that, high glycemic index diet, even if the patients do not provide too many calories per day, increases insulin synthesis which can result in increased hepatic synthesis of the insulin growth factor 1 (IGF-I) [5]. A recent study demonstrated that a chronic high dietary advanced glycation end-products could lead to a vascular dysfunction and inflammatory activation, contributing to the development of vascular complications in subjects with type 2 diabetes [6]. It is theorized that obese women with PCOS, in comparison with non-PCOS women, may face additional barriers in achieving weight loss [7] and weight loss interventions may be less effective in PCOS women compared to their non-PCOS counterparts [8]. It has been suggested that abnormalities in their appetite regulation [9] or in specific eating behaviors (higher prevalence of emotional eating) predispose them to overeating or higher consumption of high GI food items [10, 11].

The international carbohydrate quality consortium (ICQC) propose the benefits of a low GI diet for participants with hyperinsulinism [4]; however few studies have investigated or compared the effects of an energy restricted low GI diet on the reproductive, endocrine and metabolic parameters in women with PCOS to those in eumenorrheic non-hirsute women.

The aim of this study is to compare the effects of the hypocaloric LGI diet on anthropometric variables and insulin resistance in PCOS women with non-PCOS ones and to investigate the effect of this diet on the clinical and hormonal features of PCOS women.

## Methods

### Ethical approval

Written informed consent was obtained from all participants and the study was approved by the ethics committee of the Research Institute of Endocrine Sciences (approval no: 2ECRIES93/10/23). This study is registered in the Iranian Randomized Clinical Trials Registry (IRCT, code: IRCT2016092129909N1).

### Participants and setting

This study was carried out at the Reproductive Endocrinology Research Center, Research Institute for Endocrine Sciences, Shahid Beheshti University of Medical Sciences, Tehran, Iran from 2016 to 2018. One hundred and eight overweight/obese women, aged 18-40 years, including 50 PCOS and 58 non-PCOS women, eumenorrheic non-hirsute controls, were invited to participate in the present study. The PCOS group were recruited from PCOS women, attending the Reproductive Endocrinology Research Center, Shahid Beheshti University of Medical Sciences (SBUMS). PCOS was defined using Rotterdam criteria by the presence of two or more of the following: 1) Oligo- and/or anovulation, 2) Hyperandrogenemia and/or hyperandrogenism 3) Polycystic ovaries (PCO) [12]. Non-PCOS controls were recruited from women attending for their annual visit to the health care center affiliated to SBUMS. Participants were excluded if they were pregnant, breast feeding, using insulin-sensitizing agents or lipid-lowering therapies, had used contraceptive drugs during the previous 6 months, on special diets or exercise for weight loss, antihypertensive, antipsychotic or on hormonal drugs. Women with histories of any type of mental disease, chronic disease, malignancy or had participated in previous similar studies previously were also excluded.

Age and BMI levels of invited non-PCOS controls were matched with PCOS cases; to do this, participants were subdivided into < 25, 25–30 and over 30 year- old age groups, and further into BMI 25–30 and over 30 kg/m^2^ BMI groups; hence PCOS cases were categorized into six age and BMI sub-groups. Finally, a total number of 73 women, including 33 PCOS and 40 BMI and age matched non-PCOS controls agreed to participate.

### Dietary intervention

The energy restricted LGI diet was calculated for an assumed BMI of 22 kg/m^2^ and a deficit of 500 kcal (Kcal) that possibly caused an approximately 0.5 kg weight loss per week. Target macronutrient composition was 50% of energy from carbohydrate (CHO) with low and medium GI [13], 20% of energy from protein and 30% of energy from fat. A list of food items with high GI was prepared and all participants were forbidden to consume any high GI foods. A standard booklet food exchange list and eating behavior training were provided and all participants were instructed by a dietitian.

All participants were instructed to consume lean meat, whole grains, low fat dairies, non-starchy vegetables, vegetable oils and were prohibited from eating fast foods or food high in salt. In order to increase compliance with the diet, a food menu was prepared for each participant based on their energy requirement and eating habits. Fortnightly counselling visits were provided to participants to train them in recording daily dietary intakes and food compliance. To assess food compliance, a 3 day dietary food record (2 working days and one weekend day) was completed twice a month. Energy and nutrient intake of each food item was calculated using Nutritionist IV software. The US Department of Agriculture food composition table (USDA) was used as the nutrient database.

The International Table of glycemic index (GI) and glycemic load (GL) was used to calculate GI values [13]. Glycemic load was calculated by multiplying the carbohydrate content of each food per serving by the food’s GI value and dividing it by 100. In this study adherence to diet was determined as percent of energy provided from CHO, protein and fat.

### Anthropometric and biochemical assessments

Height was measured to the nearest 1 cm, without shoes in a standing position using a portable height meter measuring device. Weight was measured with a digital scale with an accuracy of 100 g. BMI was calculated by dividing weight by the square of height in meters. Waist circumference was measured using a tape at the narrowest part between the last rib and pelvic crest at the end of exhalation. Hip circumference was measured at the widest part and the waist to hip ratio was calculated. An overnight fasting venous blood sample was obtained from each participant, between the 2nd - 5th days of their spontaneous menstrual cycles and in amenorrheic women with PCOS at baseline and at weeks 12 and 24 of the intervention. It has been shown that the free androgen index (FAI) has a good correlation with free testosterone, measured by the physical separation method [14], and calculated using the formula total testosterone (nmol/L) × 100/SHBG (nmol/L). Sex hormone binding globulin (SHBG) was assessed by Electrochemilumniscence immunoassay (ECLIA), using commercial kits (Roche Diagnostics); testosterone was measured by enzyme immunoassay (EIA) using commercial kits (Diagnostic Biochemical). Fasting glucose was evaluated using enzymatic colorimetry by application of commercial kits (Pars Azmoon); and insulin measurement was obtained by ECLIA, using commercial kits (Roche Diagnostics). The inter assay coefficients of variance (CVs) were 1.1, 1. 6%, 5. 7% and 2. 7% for glucose, insulin, testosterone and SHBG, respectively. Venous blood samples were taken and centrifuged for 10 min; plasma was separated and frozen at − 80 C. After completion of the study, all biochemical analysis were performed for each participant in the same assay. Insulin resistance (IR) was estimated by the homeostasis model assessment (HOMA-IR) as a surrogate for measurement of insulin resistance according to the formula HOMA-IR = [(Fasting insulin level (mU / L) × Fasting plasma glucose (mmol / L)]/ 22.5 [15].

### Clinical measurements

After 15 min of sitting, blood pressure (BP) was measured on the left arm twice, and the average was recorded. Modified Ferriman Gallwey scoring method [16] was used to assess hirsutism by the main researcher (F.S) under supervision of a gynecologist (FRT).

Participants were asked to record their menses calendars 6 months before the commencement of study and during intervention. Menstrual cycles less than 21 days or over 35 days for at least 3 successive cycles were considered as menstrual irregularity. A change from irregular to regular cycles were considered as improvement in menstrual cyclicity.

All participants were requested to sustain their baseline physical activity. The short form of the International Physical Activity Questionnaire (IPAQ) [17] was completed every 2 weeks. This questionnaire has 7 questions about vigorous, moderate physical activity and walking time during the past week. Physical activity was calculated according to the IPAQ protocol, which scores Met level of 8 for vigorous, 4 for moderate intensity and 3. 3 for walking; total physical activity then was evaluated as follows:

Met level × days per week × minutes of activity [17].

### Statistical analysis

A sample of 60 participants (30 per group) was needed to detect a 5% weight loss in the PCOS group compared to 10% in the non-PCOS controls, with a two-sided 5% significance level, a power of 80% and loss to follow up rate of 15%.

Continuous variables were checked for normality using the one-sample Kolmogorov-Smirnoff test and normal plot; categorical variables are expressed as percentages, and are compared using Pearson’s χ^2^ test. Baseline characteristics are presented as mean ± standard deviation for normal distributed variables. Differences in baseline descriptive characteristics of study groups were explored using t-test analysis. The Mann-Whitney U test was applied to compare the baseline values of variables with skewed distributions.

The Generalized Estimation Equation (GEE) method was used to compare mean values of dietary intake and physical activity at three study time points (baseline, 12 and 24 weeks) of intervention between groups. GEE analysis was also conducted to define the effects of the energy restricted LGI diet overtime on various factors, including anthropometric, hormonal and metabolic parameters in both groups and regularity of menstrual cycles, improvement in hirsutism and acne among PCOS participants. McNemar test was used to define the changes in percentage of irregular menstrual cycles between baseline and end of intervention and logistic regression analysis was conducted to identify the impact of influencing factors on the improvement of menstrual irregularity.

## Results

**Fig. 1:**
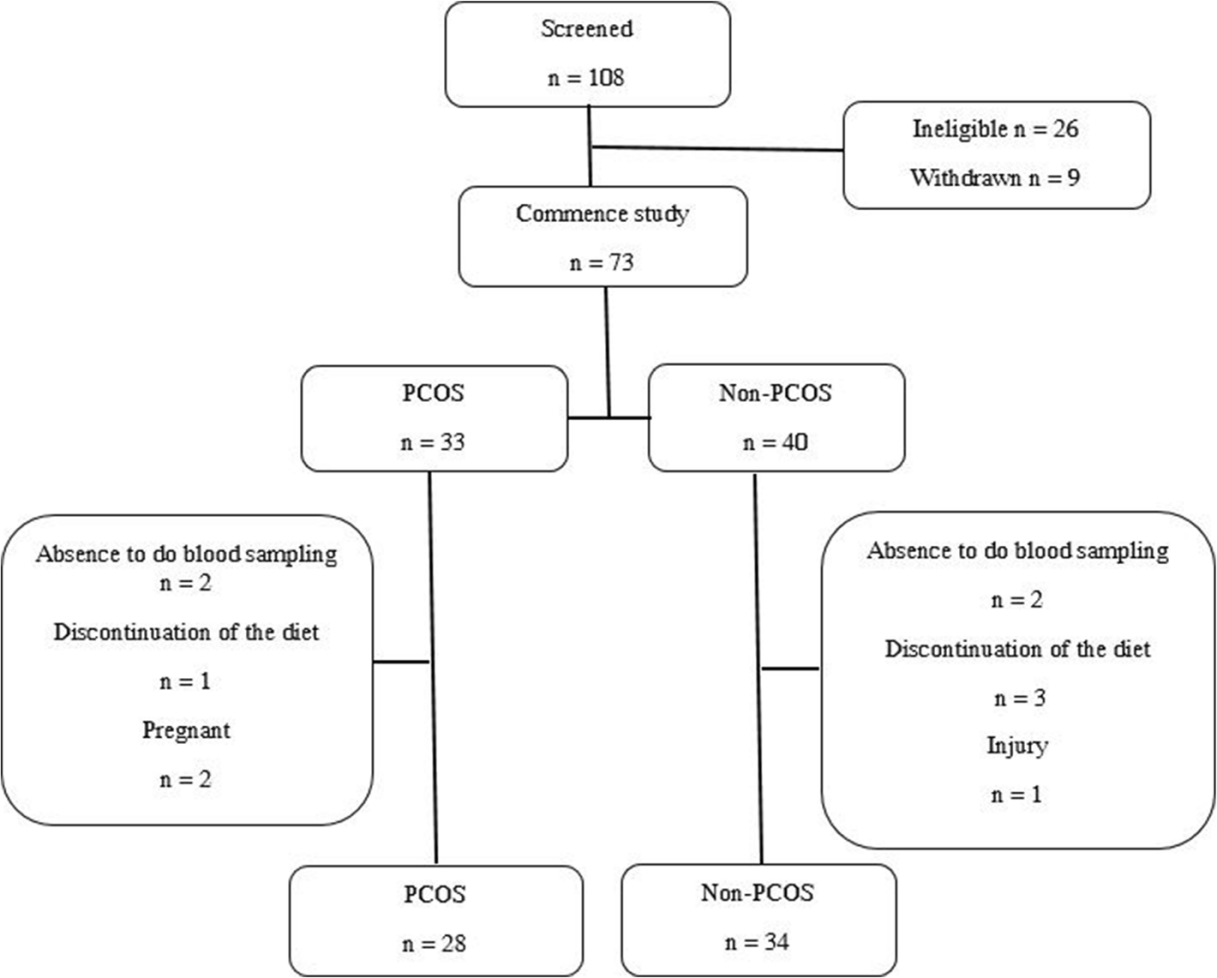
Study flow chart

Of 108 women screened for the purpose of the present study, 35 were excluded before initiation of the study (not meeting inclusion criteria, *n* = 26, refused to participate, *n* = 9) and 73 participants meeting the study inclusion criteria were enrolled in the study (PCOS = 33, non-PCOS = 40); of these 62 participants (PCOS = 28, non-PCOS = 34) completed 24-weeks of energy restricted LGI diet. The loss to follow-ups was 15% in both groups; there was no difference between the attrition in cases and controls (Fig. 1).

Both groups tolerated the dietary intervention and no adverse effects were reported. Characteristics of women with PCOS and controls are shown in Table 1. There were no statistical significant differences in demographic, anthropometric, and metabolic characteristics of study groups except for pregnancy status (*P* < 0.001), frequency of menstruation in the last 6 months (*P* < 0.001), acne status and hirsutism score (*P* < 0.001) (Table 1).

**Table 1:** Baseline characteristics of women with polycystic ovary syndrome compared to Eumenorrheic non hirsute controls

Characteristics	PCOS (*n* = 33)	Eumenorrheic non hirsute controls (40)	*P-*value
Age (years)	29. 7 ± 5. 2	30. 8 ± 4.5	0. 3
Age at menarche (years)	13.1 ± 0. 6	13.1 ± 0. 7	0. 7
Education, N (%)			
Diploma and higher	22 (78. 6%)	29 (85. 3%)	0. 4
Pregnancy status			
Never pregnant	20 (60. 6%)	4 (10%)	< 0.001
Ever pregnant	13 (39. 4%)	36 (90%)	
Weight (kg)	78.9± 12.1	76. 6± 8.9	0.37
Body mass index (BMI) (kg/m^2^)	31 ± 0.93	30.9 ± 0.5	0.9
Waist circumference (cm)	95. 4 ± 2. 2	94. 3 ± 1.1	0. 6
Hip circumference (cm)	114 ± 1. 6	111.9 ± 1. 4	0. 4
Waist to hip ratio (cm)	0.83 ± 0.009	0.84 ± 0.007	0. 7
Systolic Blood pressure (mmHg)	120.5 ± 10. 3	118. 8± 7. 3	0.46
Diastolic Blood pressure (mmHg)	77. 2± 6.9	78. 2± 4. 8	0.49
Frequency of menstruation in the last 6 months	3.46 ± 0.99	6 ± 0.1	< 0.001
Hirsutism score (FG score)	7.89 ± 5.19	1.03 ± 0.9	< 0.001
Acne, N (%)	14 (50%)	–	–
Physical activity ((Met-minute/week)	167.5± 20	147.94 ± 18.22	0.1
Energy (Kcal)	2266.9 ± 378.1	2197. 3±283. 6	0.41
Fasting blood glucose (mg/dl)	80. 8±9.9	85. 2±9.9	0.08
Fasting blood insulin (mu/L)	14. 2± 6. 2	13. 4± 7.0	0.62
HOMA-IR	2. 8±1. 3	2. 8±1.5	0.98

Data are presented as mean ± SD and number (%)

Independent t-test was for continuous variables and Pearson’s χ^2^ test for categorical ones.

*Kg* Kilogram, *cm* Centimeter, *mmHg* Millimeters of mercury, *Kcal* Kilocalories, *mg/dl* milligrams /deciliter, *mu/L* Milliunits/ liter

^*^significant difference (*P* < 0.05)

At baseline, there was no significant difference between dietary intakes and the physical activity levels of PCOS cases and controls (Table 2).

**Table 2:** Dietary intakes and physical activities of women with polycystic ovary syndrome compared to eumenorrheic non hirsute controls following 12 and 24-week of energy restricted low glycemic index diet

Variables	Baseline	*P value (Baseline)*	12 weeks	24 weeks	∆ 24–0 weeks	Within-group *P*-value^¥^	Between-group *P-*value^£^
Energy (Kcal)		0.41				< 0.001	0.54
PCOS	2266.9 ± 71.5		1317 ± 33. 3	1274.7 ± 21^a^	-992. 2±78.9		
Controls	2197. 2 ± 48. 6		1310.7 ± 31	1258.5 ± 19.1	-938. 7±50.9		
Carbohydrate (%)		0.31				< 0.001	0.47
PCOS	57. 8 ± 07		49. 7 ± 1.1	50. 6 ± 1.1	-7. 2 ± 1. 2		
Controls	58. 7 ± 0. 6		50. 2 ± 1	50.1 ± 1	-8. 6 ± 1. 3		
Carbohydrate (gram/day)		0.17				< 0.001	0.22
PCOS	307.5 ± 7. 2		146. 3 ± 8.5	156. 3± 7. 2	-151. 3 ± 11. 3		
Controls	322. 6 ± 7.9		152 ± 9.1	150.1 ± 6. 8	-172. 4 ± 9.9		
Protein (%)		0.13				< 0.001	0.82
PCOS	10.55 ± 0. 4		21. 2 ± 1. 3	20. 2 ± 1	9. 7 ± 1.1		
Controls	11. 2 ± 03		21 ± 1. 4	20. 4 ± 1^a^	9. 2 ±1		
Fat (%)		0.32^*^				0.041	0.22
PCOS	31. 7 ± 1		29 ± 0.9	29. 2 ± 0. 6	-2.5 ± 1. 2		
Controls	30 ± 0. 7		28. 8± 0.9	29. 4 ± 0. 7	-0. 6 ± 0.9		
Fiber (gram/day)		0. 8^*^				0.022	0.82
PCOS	25. 6± 2. 4		30. 2 ± 1	30. 7± 0. 6	5.1 ± 2.5		
Controls	26.5± 3		30. 2 ± 0.9	30. 8 ± 0. 7	4. 3 ± 3		
GI		0.55				< 0.001	0.47
PCOS	59.9 ± 1		41. 4 ± 2. 4	41.5 ± 2. 2	−18.5 ± 2. 3		
Controls	59 ± 1. 3		41. 3 ± 2.5	43.1 ± 1. 8	-15. 8 ± 1.9		
GL		0.17				< 0.001	0.28
PCOS	142. 4 ± 3. 8		63. 8 ± 5. 6	66.1 ± 4. 7	-76. 3 ± 5. 7		
Controls	152. 4± 6		66. 7 ± 6	66.1 ± 4	-86. 3 ± 5		
Physical activity (Met- minute_week)		0.1^*^				0.32	0. 2
PCOS	167.5± 20		170 ± 22. 2	171.57 ± 15.42	4. 2 ± 1.5		
Controls	147.9 ± 18. 2		154. 4 ± 20. 2	152 ± 13.99	5.1 ± 1. 8		

Values are mean ± SE, The Generalized estimation equation (GEE) method was used to compare the mean values at three study time points (baseline, 12th week and at 24 weeks) and between PCOS and controls

*PCOS* Polycystic ovary syndrome, *Kcal* Kilocalories, *GI* Glycemic index, *GL* Glycemic load

*Mann-Whitney U test was used to compare baseline variables between PCOS and controls

¥within-group *P*-value = effect of intervention (24-week of energy restricted low glycemic index diet) on variables; £between-group *P*-value = comparison the effect of intervention (24-week of energy restricted low glycemic index diet) on variables between PCOS and controls

At the end of 24-weeks of the energy restricted LGI diet, the observed decrease in the intakes of energy (PCOS = − 992. 2± 78.9, non-PCOS = -938. 7± 50.9 kcal/day, *p* = 0.54), carbohydrate (PCOS = − 151. 3± 11. 3, non-PCOS = − 172. 4±9.9 g/day, *p* = 0.22), fat percent (PCOS = − 2.5 ± 1. 2, non-PCOS = − 0. 6±0.9, *p* = 0.22), GI (PCOS = − 18.5± 2. 3, non-PCOS = − 15. 8±1.9, *p* = 0.47) and GL (PCOS = − 76. 3±5. 7, non-PCOS = − 86. 3±5, *p* = 0.28) were highly significant within each group, but were not significantly different between groups. Fiber (PCOS = 5.1± 2.5, non-PCOS= 4. 3± 3.0 g/day, *p* = 0.82) and percent of protein intake (PCOS = 9. 7±1.1, non-PCOS = 9. 2±1, p = 0.82) were increased in both groups but were not significantly different (Table 2).

After 24-weeks of the energy restricted LGI diet, there was a significant weight reduction within each group, when compared to baseline in the PCOS (79± 2.30 kg vs. 72.65± 2.40 kg, *p* < 0.001) and non-PCOS (76.60 ± 1.53 kg vs. 70.40 ± 1.52 kg, *p* < 0.001); there was also a significant change in BMI, compared to baseline, in the PCOS group (31 ± 0.95 kg/m^2^ vs. 28.54 ± 1 kg/m^2^, *p* < 0.001) and in the non-PCOS group (30.91 ± 0.52 kg/m^2^ vs. 28.42 ± 0.56 kg/m^2^, *p* < 0.001). Total weight reduction did not differ significantly between the two groups (− 6.70 ± 0.56 kg for PCOS group and -6. 21± 0.51 kg for non PCOS group, *p* = 0.57), with both groups achieving approximately the same percentage of weight loss (8.04% in PCOS vs. 8.09% in non-PCOS). Neither did physical activity levels in both groups differs significantly at baseline and during intervention (Table 2).

Compared to baseline, after 24-weeks of the energy restricted LGI diet, a significant reduction in insulin levels was detected in both groups of PCOS (14.25 ± 1.17 mu/L vs. 9.45 ± 1. 27 mU/L, *p* = 0.001) and non PCOS women (13.41 ± 1. 2 mU/L vs. 9.64 ± 0.78 mU/L, *p* = 0.001), a reduction however not statistically significant between PCOS and non PCOS women (− 4.77 ± 1.57 mU/L in PCOS vs. -3.77 ± 1. 3 mu/L in non-PCOS controls, *p* = 0.59). No significant difference in changes of HOMA-IR (*p* = 0.83), and fasting blood glucose (*p* = 0.73) were observed during follow up in the PCOS, compared to the non-PCOS controls.

Although after the 24-week energy restricted LGI diet, there was no significant difference in changes of systolic blood pressure between the two groups (*P* = 0.81); patients with PCOS had a higher reduction in their diastolic blood pressure compared to those of non-PCOS (*P* < 0.001) (Table 3).

**Table 3:** Results of the generalized estimation equation models showing the effect of 12 and 24-week of energy restricted low glycemic index diet on anthropometric and metabolic parameters in women with polycystic ovary syndrome compared to eumenorrheic non hirsute controls

parameters	Variables	Baseline	12 weeks	24 weeks	∆ 24–0 weeks	Within-group *P*-value^¥^	Beta Coef.	95% Confidence Interval	Between-group P-value^£^
Weight (kg)	PCOs	79± 2.30	75.34± 2.30	72.65± 2.40	-6.70 ± 0.56	< 0.001	2.50	(− 2.80, 7.80)	0.53
	Controls	76.60 ± 1.53	73.01 ± 1.52	70.40 ± 1.52	-6. 21±0.51	< 0.001	Reference		
	Time						-3.25	(− 3.72, − 2.80)	< 0.001
	PCOs* Time						-. 20	(− 0.90,0.50)	0.57
	Controls*Time						Reference		
BMI (kg/m^2^)	PCOs	31.00 ± 0.95	29.58 ± 0.94	28.54 ± 1.00	-2.62 ± 0. 20	< 0.001	0.24	(− 1.80, 2. 27)	0.81
	Controls	30.91 ± 0.52	29.47 ± 0.53	28.42 ± 0.56	-2.50 ± 0. 20	< 0.001	Reference		
	Time						−1.19	(−1.33, − 1.05)	< 0.001
	PCOs* Time						−0.1	(− 0.30, 0. 11)	0.36
	Controls*Time						Reference		
WC (cm)	PCOs	95.48± 2.26	91.25± 2.36	88.74± 2.41	-7.10 ± 0.77	< 0.001	1.40	(−3.66, 6.45)	0.58
	Controls	94.31 ± 1. 20	90.38 ± 1. 20	87.68 ± 1.26	-6.63 ± 0.62	< 0.001	Reference		
	Time						-2.86	(− 3.43, − 2.30)	< 0.001
	PCOs* Time						− 0.24	(− 1.08, 0.60)	0.57
	Controls*Time						Reference		
SBP (mmHg)	PCOs	120.50 ± 1.94	117.57 ± 1.83	119.92± 3.47	−0.63± 3.32	0.67	0.97	(−5.94, 7.89)	0.78
	Controls	118.79 ± 1.26	117.15 ± 1.34	117.32 ± 1.51	−1.80 ± 0.52	< 0.001	Reference		
	Time						−0.87	(− 2.72,0.98)	0.36
	PCOs* Time						0.33	(−2.36, 3.02)	0.81
	Controls*Time						Reference		
DBP (mmHg)	PCOs	77.18 ± 1.30	76.53 ± 1.17	76.00 ± 1. 12	−1.18 ± 0.65	0.03	−0.02	(−2.86, 2.83)	0.98
	Controls	78.25 ± 0.85	78.88 ± 0.81	78.73 ± 0.79	0.41 ± 0. 21	0.05	Reference		
	Time						0.37	(0.05,0.69)	0.02
	PCOs* Time						−0.99	(− 1.46, − 0.52)	< 0.001
	Controls*Time						Reference		
FBG (mg/dl)	PCOs	80.86 ± 1.87	82.75 ± 1.62	83. 11±1.97	2.25± 2. 27	0.34	-4.48	(− 10.93, 1.98)	0.17
	Controls	85.23 ± 1.69	85.60± 2.35	86.23 ± 1. 21	1.00 ± 1.90	0.61	Reference		
	Time						0.51	(−1.41, 2.43)	0.60
	PCOs* Time						0.49	(−2.40, 3.38)	0.73
	Controls*Time						Reference		
Fasting Insulin (mu/L)	PCOs	14.25 ± 1.17	12. 11±1.48	9.45 ± 1. 27	-4.77 ± 1.57	0.001	1.45	(−3.38, 6.28)	0.55
	Controls	13.41 ± 1. 20	10.84 ± 1.50	9.64 ± 0.78	-3.77 ± 1.30	0.001	Reference		
	Time						−1.88	(− 3.30, − 0.47)	0.009
	PCOs* Time						− 0.57	(− 2.68, 1.54)	0.59
	Controls*Time						Reference		
HOMA_IR	PCOs	2.82 ± 0.24	2.48 ± 0.33	1.96 ± 0.31	− 0.83 ± 0.33	0.001	0.06	(− 0.10, 1. 12)	0.91
	Controls	2.82 ± 0.26	2.26 ± 0.34	2.03 ± 0.16	−0.79 ± 0.28	0.001	Reference		
	Time						−0.40	(−0.70, − 0.09)	0.01
	PCOs* Time						−0.05	(− 0.51, 0.41)	0.83
	Controls*Time						Reference		

Values are mean ± SE; The generalized estimation equation (GEE) method was used to estimate the effect of 12 and 24-week of energy restricted low glycemic index diet on anthropometric and metabolic parameters in women with polycystic ovary syndrome versus eumenorrheic non hirsute controls

*BMI* Body mass index, *WC* Waist circumference, *SBP* Systolic blood pressure, *mmHg* Millimeters of mercury, *DBP* Diastolic blood pressure, *mg/dl* Milligrams /deciliter, *mu/L* Milliunits/ liter, *FBG* Fasting blood glucose, *HOMA_IR* Homeostatic model assessment

^¥^within-group *P-*value = effect of intervention (24-week of energy restricted low glycemic index diet) on anthropometric and metabolic parameters; ^£^between-group *P*-value = comparison the effect of intervention (24-week of energy restricted low glycemic index diet) on anthropometric and metabolic parameters in women with polycystic ovary syndrome versus eumenorrheic non hirsute controls

**Fig. 2:**
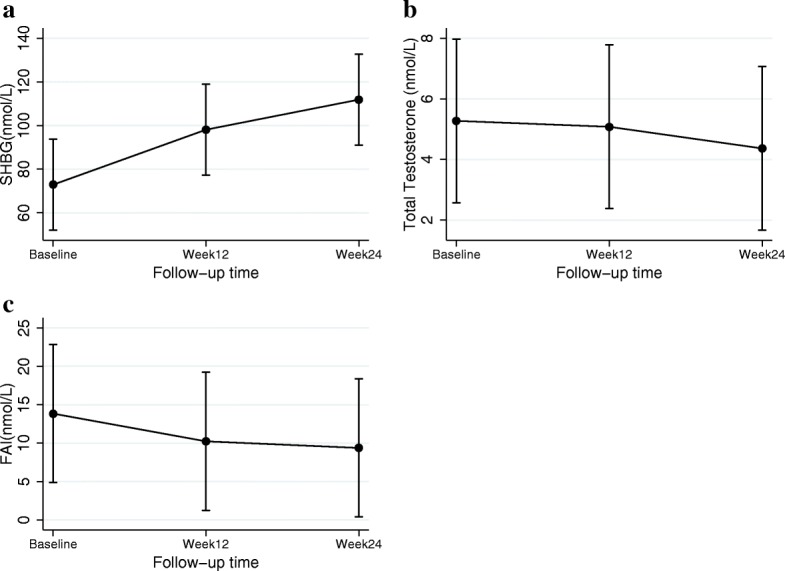
**a** Mean (±SEM) Sex hormone binding globulin (SHBG) concentration (nanomole/liter, nmol/L), **b** Total testosterone (nmol/L), **c** Free androgen Index (FAI) at baseline, 12 and 24 weeks of intervention

For PCOS participants, an increase in SHBG (38.98± 11.02 nmol/L, *P* < 0.001) (Fig. 2.a) and a reduction in total testosterone and FAI were reported from baseline to week 24 of the study (total testosterone − 0.91 ± 0.33 nmol/L *P* = 0.006, FAI = − 4.47 ± 1.10, *P* < 0.001) (Fig. 2.b, c). There was a negative association between the amount of increase in SHBG and weight loss (r = − 0.41, *P* = 0.03).

In the PCOS group, at baseline, the mean intervals of menstrual cycles were 51.36 ± 15. 12 days and the mean menstrual frequency per year was 3.46 ± 0.99; after 24 weeks of the energy restricted diet these parameters were improved to 42.59 ± 16.34 days, *P* < 0.001 and 4.0 ± 1.37 (*P* = 0.01), respectively. At baseline, of 28 PCOS women (85. 7%), 24 had menstrual irregularities; after 24 weeks of the energy restricted diet, 80% of women reported regular menstrual cycles (*P* < 0.001). Logistic regression analysis showed that odds of regularity of menstrual cycles were increased by reduction in BMI and HOMA-IR Z-scores (OR = 1.09, *p* = 0.04 and OR= 3.76, *p* = 0.04, respectively). During the study, Ferriman–Gallwey score decreased from 7.89 ± 5.19 to 6.62± 4. 6 (*P* = 0.001) at week 24 of intervention. At baseline, 50% of participants with PCOS had acne and a 32.1% reduction (*p* = 0.004) in acne was reported at the end of follow up. At baseline, among PCOS women with acne, distributions of acne severity (categorized as without acne, mild, medium, and severe) were 50, 21. 4%, 25, and 3. 6%, respectively; values improved to 82.1, 10. 7%, 3. 6%, and 0% after 24 weeks of intervention, respectively (*p* < 0.001).

## Discussion

The present study demonstrates that an energy restricted LGI diet has similar beneficial effects on the anthropometric and metabolic characteristics of overweight women with and without PCOS. Moreover, in PCOS women, both the regularities of menstrual cycles and the clinical and biochemical features of hyperandrogenism were improved after 6 months of implementing this diet.

Although earlier studies suggest that PCOS is often associated with an increased risk of metabolic disorders, in particular obesity, it is unclear whether the energy restricted LGI diet can improve these disorders as well as non-PCOS women [3, 4]. Our study findings are in contrast with those of studies reporting that women with PCOS may face difficulties in achieving weight loss [8], due to metabolic issues [18] or the emotional eating problems [17] that accompany this disorder [11]. Furthermore, it has been reported that decreased basal metabolic rate (BMR) or post-prandial thermogenesis [18] make PCOS women susceptible to obesity [19], although other studies have shown that there was no difference in BMR and post prandial thermogenesis in women with or without PCOS [20].

There is some evidence that the LGI diet delays absorption of carbohydrates and improves metabolic pathways and insulin resistance [21]. Studies conducted on obese women in general populations demonstrated that a LGI diet can induce decrease in appetite and food intakes, and increased fat oxidation, decreased lipogenesis, accumulation of fat and insulin secretion [22]. Majority of women with PCOS show a marked compensatory hyperinsulinema after carbohydrate ingestion; there may be specific advantages of LGI diets for this group. Some studies report that using the LGI diet in PCOS women may improve metabolic features and insulin resistance [23], although it has been assumed that obese women with PCOS had more difficulties in weight loss, compared to non-PCOS ones, a hypothesis that dissuades PCOS women from adherence to this diet. In the present study, we found that both PCOS women and non-PCOS controls have similar improvement in weight loss, fasting insulin and HOMA. Previous studies show conflicting results regarding the effect of weight loss on IR and fasting glucose levels; the energy restricted diet induced a reduction in fasting insulin and IR in women with a history of gestational diabetes [24] and in patients with syndrome X [25, 26]. In contrast, Herriot et al. [24] reported that the LGI diet induces a decrease in fasting glucose and weight without alteration in insulin levels. In agreement with our results, Moran et al. [27] reported similar reductions in fasting insulin and weight in PCOS women and non-PCOS controls. Some studies report an increase in prevalence of hypertension among PCOS women compared to the general population, regardless of their weight [28].

In the present study, women with PCOS, compared to their non-PCOS counterparts, have similar systolic and diastolic blood pressures at baseline. After the 24-week energy restricted LGI diet, a subtle reduction in systolic blood pressure was observed in both PCOS women and non-PCOS controls, although this change was not clinically important. This finding is consistent with those of another study, demonstrating that a low GI diet did not cause a reduction in blood pressure [29].

We found that PCOS women had a significant reduction in serum levels of testosterone, FAI and an increase in SHBG, findings in agreement with another study that also showed the beneficial effects of weight loss on reproductive hormones [30]. This study revealed significant alterations in BMI and HOMA. Improvement in insulin resistance through weight loss or use of sensitizing insulin drugs leads to decrease in hyperandrogenemia. In comparison with non-PCOS women, the theca cells of PCOS women are more sensitive to insulin. Insulin augments the effect of LH, thereby increasing androgen secretion due to the synergistic effect of LH and insulin. Furthermore insulin decreases hepatic SHBG production and increases bioavailable testosterone. Therefore, in obese women with PCOS, free androgen levels are increased and insulin-like growth factor binding protein-1(IGFBP-1) is decreased. Weight loss causes reduction in insulin levels and enhancement of IGFBP-1 and inhibits cytochrome P450 17 system, thereby decreasing androgen production [31].

Previous studies show the benefits of restriction of calories and the resulting weight loss in improving ovarian function and menstrual regularity [23, 32]. In our study 80% of women with irregular menstrual cycles at baseline achieved regular menstruation after the 24-week energy restricted LGI diet. Our results demonstrated that a greater reduction in BMI and HOMA may be significantly associated with improvement of menstrual regularities. Greater reductions in HOMA and BMI in women with improved menstrual regularities confirm the key role of insulin resistance and obesity in pathogenesis of PCOS. A greater weight loss and reduction in HOMA and better endocrine profile in women with restored menstrual regularity was also demonstrated in some [32, 33] but not all [3]; one study showed that weight, abdominal fat loss and insulin resistance were the same in women with and without improvement of menstrual cycles [34].

In this study, significant improvements in mean FG-scores were determined after 24 weeks of intervention, a result in line with those of some life style modification studies [30, 32, 33]; however, other studies suggest no effect of weight reduction on FG-scores or hirsutism [34], a controversy that could partly be explained by short duration of intervention, since the intervention effects on hirsutism need much longer follow ups. Our study had a long-term follow-up, which could be adequate for accurate conclusion.

In the present study, in agreement with others, we found a decrease in the occurrence and severity of acne [35]. Recent studies suggest that dietary factors, specifically glycemic load are involved in the pathogeneses of acne. It is well documented that there is a significant association between acne and obesity. In addition, hyperinsulinism, a prevalent metabolic disorder in obese women, increases bioavailability of androgen, IGF-1 and lipogenesis of sebaceous cells [36].

### Strengths and limitations

Our study strengths include having a control group and simultaneous medication. Another strength of this study is high levels of adherence to the dietary plan and dropout rate of 15%; as this study implemented a diet based on participants’ dietary habits, increased number of follow ups, and emphasized modification of eating behavior, like omitting non-hunger-eating, rapid eating, food with high energy density, and consumption of food while watching TV, all of which improved the compliance of our participants. Our study had also assessments of physical activity before and during intervention, which may prevent the potential influencing role of different physical activity status on the beneficial effect of LGI diet in terms of clinical and endocrine variables. Age and BMI matching of cases and controls can prevent all the biases that could have arisen from differences in age and BMI.

Our study has its limitations as well; we used self-reporting questionnaires, relying on participants’ reports on compliance with their prescribed diet. Adiposity and inflammatory markers were not assessed. We did not also assess the lipid profiles of PCOS patients; however, since previous studies showed that lipid lowering therapies can improve PCOS clinical and ovarian dysfunction abnormalities [37–39], we excluded these patients from the study. We have used HOMA-IR as a surrogate marker for assessing IR. Our study did not have enough power for comparison of various PCOS phenotypes, since this comparison was not the initial objective of the present study.

## Conclusions

Study results demonstrate that the energy restricted LGI diet induces equally beneficial decrease in weight and insulin resistance in women with or without PCOS, by confirming the effect of energy restricted LGI diet in enhancement of endocrine and clinical variables in PCOS women. Improvement of menstrual irregularities in women with PCOS was associated with greater weight loss and improved HOMA. The efficacy of LGI diet in improving IR, hyperandrogenism, hirsutism, acne, menstrual irregularities in addition to its high dietary compliance make the LGI diet an optimal dietary choice for women with PCOS. For better comparison of the effects of this diet in PCOS women with their non PCOS counterparts, larger clinical trials with sufficient number of participants in each PCOS phenotype and measurements of other adiposity and body composition markers is highly recommended.

## Data Availability

The datasets used and/or analysed during the current study available from the corresponding author on reasonable request.

## Abbreviations

BMI: Body mass index
BP: Blood pressure
CV: Coefficients of variance
ECLIA: Electrochemilumniscence immunoassay
EIA: Enzyme immunoassay
FAI: Free androgen index
GEE: Generalized Estimation Eq.
GI: Glycemic index
GL: Glycemic load
HOMA-IR: Homeostasis model assessment
ICQC: International carbohydrate quality consortium
IGF-I: Insulin growth factor 1
IPAQ: International Physical Activity Questionnaire
LGI: Low glycemic index
LH: Luteinizing hormone
PCOS: Polycystic ovary syndrome
SHBG: Sex hormone binding globulin

## References

[1] Skiba MA, Islam RM (2018). Bell RJ^1^, Davis SR. understanding variation in prevalence estimates of polycystic ovary syndrome: a systematic review and meta-analysis. Hum Reprod Update.

[2] Lim SS, Norman RJ, Davies MJ, Moran LJ (2013). The effect of obesity on polycystic ovary syndrome: a systematic review and meta-analysis. Obes Rev.

[3] Thomson RL, Buckley JD, Noakes M, Clifton PM, Norman RJ, Brinkworth GD (2008). The effect of a hypocaloric diet with and without exercise training on body composition, cardiometabolic risk profile, and reproductive function in overweight and obese women with polycystic ovary syndrome. J Clin Endocrinol Metab.

[4] Augustin LS, Kendall CW, Jenkins DJ, Willett WC, Astrup A, Barclay AW, Björck I, Brand-Miller JC, Brighenti F, Buyken AE (2015). Glycemic index, glycemic load and glycemic response: An International Scientific Consensus Summit from the International Carbohydrate Quality Consortium (ICQC). Nutr Metab Cardiovasc Dis.

[5] Szczuko M, Zapałowska-Chwyć M, Maciejewska D, Drozd A, Starczewski A, Stachowska E (2016). High glycemic index diet in PCOS patients. The analysis of IGF I and TNF-α pathways in metabolic disorders. Med Hypotheses.

[6] Di Pino A, Currenti W, Urbano F, Scicali R, Piro S, Purrello F, Rabuazzo AM (2017). High intake of dietary advanced glycation end-products is associated with increased arterial stiffness and inflammation in subjects with type 2 diabetes. Nutr Metab Cardiovasc Dis.

[7] Moran L, Gibson-Helm M, Teede H, Deeks A (2010). Polycystic ovary syndrome: a biopsychosocial understanding in young women to improve knowledge and treatment options. J Psychosom Obstet Gynaecol.

[8] Teede HJ, Joham AE, Paul E, Moran LJ, Loxton D, Jolley D, Lombard C (2013). Longitudinal weight gain in women identified with polycystic ovary syndrome: results of an observational study in young women. Obesity.

[9] Japur CC, Diez-Garcia RW, Oliveira Penaforte FR (2014). Imbalance between postprandial ghrelin and insulin responses to an ad libitum meal in obese women with polycystic ovary syndrome. Reprod Sci.

[10] Shishehgar F, Tehrani FR, Mirmiran P, Hajian S, Baghestani AR, Moslehi N (2016). Comparison of dietary intake between polycystic ovary syndrome women and controls. Global J Health Sci.

[11] Jeanes Y, Barr S, Sharp K, Reeves S (2010). Eating behaviours and BMI in women with polycystic ovary syndrome. Proc Nutr Soc.

[12] The Rotterdam EA-SPCWG Revised (2004). 2003 Consensus on diagnostic criteria and long-term health risks related to polycystic ovary syndrome. Fertil Steril.

[13] Foster-Powell K, Holt SH, Brand-Miller JC (2002). International table of glycemic index and glycemic load values. Am J Clin Nutr.

[14] Rosner W, Auchus RJ, Azziz R, Sluss PM, Raff H (2007). Position statement: utility, limitations, and pitfalls in measuring testosterone: an Endocrine Society position statement. J Clin Endocrinol Metab.

[15] Matthews DR, Hosker JP, Rudenski AS, Naylor BA, Treacher DF, Turner RC (1985). Homeostasis model assessment: insulin resistance and beta cell function from fasting plasma glucose and insulin concentrations in man. Diabetologia.

[16] Hatch R, Rosenfield RL, Kim MH, Tredway D (1981). Hirsutism: implications, etiology, and management. Am J Obstet Gynecol.

[17] International Physical Activity Questionnaire (2005). Guidelines for Data Processing and Analysis of the International Physical Activity Questionnaire - Short and Long Forms.

[18] Robinson S, Chan SP, Spacey S, Anyaoku V, Johnston DG, Franks S (1992). Postprandial thermogenesis is reduced in polycystic ovary syndrome and is associated with increased insulin resistance. Clin Endocrinol.

[19] Blay SL, Aguiar JV, Passos IC (2016). Polycystic ovary syndrome and mental disorders: a systematic review and exploratory meta-analysis. Europsychiatr Dis Treat.

[20] Churchill SJ, Wang ET, Bhasin G, Alexander C, Bresee C, Pall M, Azziz R, Mathur R, Pisarska MD (2015). Basal metabolic rate in women with PCOS compared to eumenorrheic controls. Clin Endocrinol.

[21] Radulian G, Rusu E, Dragomir A, Posea M (2009). Metabolic effects of low glycaemic index diets. Nutr J.

[22] Venn BJ, Green TJ (2007). Glycemic index and glycemic load: measurement issues and their effect on diet-disease relationships. Eur J Clin Nutr.

[23] Marsh KA, Steinbeck KS, Atkinson FS, Petocz P, Brand-Miller JC (2010). Effect of a low glycemic index compared with a conventional healthy diet on polycystic ovary syndrome. Am J Clin Nutr.

[24] Tiikkainen M, Bergholm R, Vehkavaara S, Rissanen A, Hakkinen AM, Tamminen M, Teramo K, YkiJarvinen H (2003). Effects of identical weight loss on body composition and features of insulin resistance in obese women with high and low liver fat content. Diabetes.

[25] Reaven G, Segal K, Hauptman J, Boldrin M, Lucas C (2001). Effect of orlistat assisted weight loss in decreasing coronary heart disease risk in patients with syndrome X. Am J Cardiol.

[26] Herriot AM, Whitcroft S, Jeanes Y (2008). An retrospective audit of patients with polycystic ovary syndrome: the effects of a reduced glycaemic load diet. J Hum Nutr Diet.

[27] Moran LJ, Noakes M, Wittert GA, Clifton PM, Norman RJ (2012). Weight loss and vascular inflammatory markers in overweight women with and without polycystic ovary syndrome. Reprod Biomed.

[28] Lo JC, Feigenbaum SL, Yang J, Pressman AR, Selby JV, Go AS (2006). Epidemiology and adverse cardiovascular risk profile of diagnosed polycystic ovary syndrome. J Clin Endocrinol Metab.

[29] Rouhani MH, Kelishadi R, Hashemipour M, Esmaillzadeh A, Azadbakht L (2013). The effect of low glycemic index diet on body weight status and blood pressure in overweight adolescent girls: a randomized clinical trial. Nutr Res Pract.

[30] Phy JL, Pohlmeier AM, Cooper JA, Watkins P, Spallholz J, Harris KS, Berenson AB, Boylan M (2015). Low starch/low dairy diet results in successful treatment of obesity and co-morbidities linked to polycystic ovary syndrome (PCOS). Obes Weight Loss Ther.

[31] Diamanti-Kandarakis E, Dunaif A (2012). Insulin resistance and the polycystic ovary syndrome revisited: an update on mechanisms and implications. Endocr Rev.

[32] Ornstein RM, Copperman NM, Jacobson MS (2011). Effect of weight loss on menstrual function in adolescents with polycystic ovary syndrome. J Pediatr Adolesc Gynecol.

[33] Lass N, Kleber M, Winkel K, Wunsch R, Reinehr TH (2011). Effect of lifestyle intervention on features of polycystic ovarian syndrome, metabolic syndrome, and intima-media thickness in obese adolescent girls. J Clin Endocrinol Metab.

[34] Moran LJ, Noakes M, Clifton PM, Tomlinson L, Norman RJ (2003). Dietary composition in restoring reproductive and metabolic physiology in overweight women with polycystic ovary syndrome. J Clin Endocrinol Metab.

[35] Kwon HH, Yoon JY, Hong JS, Jung JY, Park MS, Suh DH (2012). Clinical and histological effect of a low Glycaemic load diet in treatment of acne vulgaris in Korean patients: a randomized, controlled trial. Acta Derm Venereol.

[36] Melnik BC, John SM, Plewig G (2013). Acne: risk Indicator for increased body mass index and insulin resistance. Acta Derm Venereol.

[37] Seyam E, Hefzy E (2018). Long-term effects of combined simvastatin and metformin treatment on the clinical abnormalities and ovulation dysfunction in single young women with polycystic ovary syndrome. Gynecol Endocrinol.

[38] Seyam E, Al Gelany S, Abd A, Ghaney A, Mohamed MAA, Youseff AM, Ibrahim EM, Khalifa EM, Hefzy E (2018). Evaluation of prolonged use of statins on the clinical and biochemical abnormalities and ovulation dysfunction in single young women with polycystic ovary syndrome. Gynecol Endocrinol.

[39] Scicali R, Di Pino A, Ferrara V, Urbano F, Piro S, Rabuazzo AM, Purrello F (2018). New treatment options for lipid-lowering therapy in subjects with type 2 diabetes. Acta Diabetol.

